# Observational studies: Ambient air pollution and hospitalization for RA-ILD in a heavily polluted city in China

**DOI:** 10.1097/MD.0000000000029309

**Published:** 2022-05-20

**Authors:** Baojin Liu, Guangzhi Sun, Ying Liu, Yanfeng Hou

**Affiliations:** a Department of Rheumatology, Central Hospital Affiliated to Shandong First Medical University, Jinan, China; b Department of Orthopedic Surgery, The First Affiliated Hospital of Shandong First Medical University & Shandong Provincial Qianfoshan Hospital, Shandong Key Laboratory of Rheumatic Disease and Translational medicine; c Department of Rheumatology, The Affiliated Hospital of Shandong University of Traditional Chinese Medicine, Jinan, China; d Department of Rheumatology and Autoimmunology, The First Affiliated Hospital of Shandong First Medical University & Shandong Provincial Qianfoshan Hospital, Shandong Key Laboratory of Rheumatic Disease and Translational medicine, Shandong medicine and Health Key Laboratory of Rheumatism. No. 16766 Jingshi Road, Jinan, Shandong, China.

**Keywords:** air pollution, hospital admission, interstitial lung disease: rheumatoid arthritis, time-series analysis

## Abstract

Little is known within the medical community about the impact of air pollution on hospital admissions due to rheumatoid arthritis associated with interstitial lung disease (RA-ILD). Our research aimed to explore whether there is a correlation and to estimate how the association was distributed across various lags in Jinan, China.

The relationships between ambient air pollutant concentrations, including PM_2.5_, PM_10_, sulfur dioxide (SO_2_), ozone (O_3_), and nitrogen dioxide (NO_2_), and monthly hospitalizations for RA-ILD were studied by employing a general linear model with a Poisson distribution. This time-series study was performed from January 1^st^, 2015 to December 31^st^, 2019.

In the 5-year study, there were 221 hospitalizations for RA-ILD in Jinan city. The levels of PM_2.5_, PM_10_, SO_2_, and NO_2_ were significantly related to the number of admissions for RA-ILD. PM_2.5_, PM_10_, and SO_2_ showed the most significant effect on the month (lag 0), and NO_2_ was most related to RA-ILD at a lag of two months (lag 2). The monthly admissions of RA-ILD increased by 0.875% (95% CI: 0.375–1.377%), 0.548% (95% CI: 0.148–0.949%), 1.968% (95% CI: 0.869–3.080%), and 1.534% (95% CI: 0.305–2.778%) for each 10 μg/m^3^ increase in PM_2.5_, PM_10_, SO_2_ and NO_2_, respectively.

This study might add more detailed evidence that higher levels of PM_2.5_, PM_10_, SO_2_ and NO_2_ increase the risk of hospitalizations for RA-ILD. Further study of the role of air pollution in the pathogenesis of RA-ILD is warranted.

## 1 Introduction

Rheumatoid arthritis (RA) is a disease related to chronic autoimmunity that has various systemic features and immune-mediated multiorgan dysfunction, characterized by symmetrical arthritis and synovitis. The common extra-articular organs involved include the cardiovascular system, lung, kidney, blood system, etc., in approximately 50% of patients with RA.^[1,2]^ Interstitial lung disease (ILD) is the main manifestation of lung involvement, and approximately 5–10% of all patients with RA will suffer clinically significant ILD. ^[3,4]^

Compared with patients who have RA without ILD, patients with RA-ILD have a three-fold increase in mortality. This means that RA-ILD has a poor prognosis, as reported.^[3]^ ILD dramatically influence patients’ lives with an average survival of just 3 years from diagnosis of RA-ILD, which accounting for 10%-20% of all deaths associated with RA.^[3,5,6]^ The underlying etiologies of RA-ILD are unknown. Early studies have reported that risk factors leading to RA-ILD consist of increased age, male sex, smoking, higher RA disease activity, and seropositivity for RA autoantibodies.^[3,4,7,8,9]^ Additionally, current research has shown that atmospheric pollutants are risk factors for both RA and ILD of known or unknown etiology, although the mechanism is unclear. ^[10,11]^ However, little correlational research has been performed between atmospheric pollutants and ILD in RA.

Air pollution is a risk for the occurrence of negative respiratory results. Air pollutants of particulate matter (PM_2.5_, a pollutant with a diameter not more than 2.5 μm), PM_10_ (a pollutant with a diameter not more than 10 μm), and NO_2_, SO_2_, and O_3_ which can deposit deeply in the airways and alveoli causing oxidative stress, inflammation and tissue injury.^[12,13]^ Research has shown that environmental exposure factors may activate innate immune cells, alter the pulmonary microenvironment and activate a cascade of pro-inflammatory and immunity-related responses that eventually cause ILD or other models of lung involvement in patients with RA. ^[14–16]^ This suggests that environmental factors are crucial during the occurrence of RA-ILD. We hypothesized that exposure to air pollution would be associated with RA-ILD.

Jinan is the economic and population center of Shandong Province, which is considered one of the regions in China with the most severe air pollution problem. There is no previous survey regarding the relationships between major air pollutants and RA-ILD in China, and we conducted time-series research to analyze the associations between air pollutants and hospitalization for RA-ILD for the first time in Jinan, China.

## 2 Materials and methods

### 2.1 Study area

Jinan is the political center of Shandong Province and among China's most polluted cities. Jinan has a distinctive topography surrounded by mountains, and the surrounding terrain is higher than the urban area, which shows a basin state. Such a topographic feature is more likely to form a “temperature inversion” phenomenon, contributing to the pollutants not being discharged and spreading outward, which can easily lead to the accumulation of air pollutants.

As the economy has soared and leveled off, the population has significantly grown and traffic has become more congested, Jinan has been plagued by serious problems of air pollution in recent years. The discharge of automobile exhaust, pollutants from heavy industrial enterprises and living sources and the combustion of coal in urban areas are all the primary sources of air pollutants, and they are excessively concentrated in urban areas with poor diffusion conditions. These situations have caused a considerable burden on pollution control and environmental protection in Jinan. In addition, the residents of Jinan require heating in winter, and coal-fired heating increases pollution emissions. At the same time, low temperatures and low wind levels are not conducive to the diffusion and dilution of pollutants. All these reasons have led to severe, frequent haze incidents in Jinan in recent years. The research area is the entire Jinan region in my research.

### 2.2 Air pollution data

Monthly average data on the concentration of ambient air pollutants were acquired from 14 fixed air quality monitoring stations situated in Jinan, Shandong Province, China, during the duration of the research period from January 1^st^, 2015, to December 31^st^, 2019. The data provide the daily and monthly maximum, minimum, and mean concentrations for PM_2.5_, PM_10_, SO_2_, NO_2_, and O_3_. Those 14 ambient air quality monitoring stations are distributed all over Jinan and obtain air pollution measurements every day. After the calculation of the daily average of each contamination from the 14 stations, the monthly mean levels of these contaminations are analyzed. All the data collected were calculated and analyzed by the Environmental Protection Authority (EPA) in Jinan city.

### 2.3 Hospital admission data

Records of hospital admission were collected from the computerized database from the Hospital Information System (HIS) of The First Affiliated Hospital of Shandong's First Medical University in Shandong Province. The coverage of this hospital is the entire Jinan.The records collected include information on sex, age, admission date, diagnosis, the address of residence, complications and other related data of patients admitted due to RA-ILD during our research period in Jinan city. All the people with RA-ILD selected to be the research subjects had to meet the diagnostic criteria for RA-ILD. Inclusion criteria included patients fulfilling the revised 1987 ACR criteria for RA ^[17]^ and the detection of ILD by high-resolution CT (HRCT). At least one rheumatologist assessed the presence of ILD on the basis of the CT reports. The reports were issued by one or two radiologists who had expertise in pulmonary CT and assessing ILD. Therefore, the data were sufficiently reliable and accurate for use in our research. We limited our study to patients who had resided in Jinan for a long time to ensure the accuracy of the regional study, and we excluded patients who were rehospitalized within one month to limit exposure misclassifications. In addition, all subjects consented to study participation after receiving notification and study information, and all aspects of the study were approved by the ethics committee of Shandong First Medical University.

## 3 Methods

In this research, the correlation between the mean concentrations of ambient air pollutants and hospitalizations due to RA-ILD was analyzed by employing the generalized additive model (GAM) with the Poisson distribution as the link function, which has reliable and satisfactory performance in previous studies.^[18,19]^ In this research, the generalized additive model of the time series can be used to explore the “lag effect” and “cumulative lag effect” on the basis of evaluating the correlation between various atmospheric pollutants and the number of patients admitted to the hospital.

Each pollutant concentration is put into the basic model as a continuous variable, and a single-pollutant model is established. The generalized additive model with Poisson distribution as the link function was employed to analyze the relationship between the concentration of various air pollutants and the number of hospitalizations for RA-ILD, the weight of which equals the hospital admission counts on that day. After adjusting for the confounders, including the long-term trend, temperature, and relative humidity, the association between them was estimated using the excess risk (ER) and 95% confidence interval (CI). Positive ER values indicated the percentage increase (%) in RA-ILD monthly admissions for an increase in pollutant concentration. Negative ER values indicate that there is a negative effect or no correlation between air pollutants and hospital admissions. All statistical analyses were conducted with R package software.

In addition, the number of inpatients with the disease may change immediately after the change in exposure factors, or there may be a gradual change in response after a certain period of exposure, that is, a lag effect. Considering the existence of a lag effect, we recorded the month of air pollutant measurement as lag time zero (lag 0), a lag of one month as lag 1, and so on. We also conducted sensitivity analyses to demonstrate the effect of air pollutants on the disease under different cumulative lag days to verify the stability of the results. A cumulative lag of one month is lag01, and a cumulative lag of two months is lag02. The derived GAM equation is as follows:


(1) $$Log\left[E\left(Y_{t}\right)\right]=\alpha +\beta_{1}C_{i}+s(Time,df)+s(RH,df)+s(Temp,df)$$

where *Y*_*t*_ shows the number of patients admitted on day *t*, and *E (Y*_*t*_*)* indicates the estimated number of patients on that day. β_1_ represents the regression coefficient, *s* represents the spline smoothing function, and C_i_ represents the average pollutant concentration of the day or lag (i) month. *Time* represents the long-term trend, *RH* represents monthly average relative humidity, and *Temp* represents monthly average temperature.

The value for the degrees of freedom (*df*) of the long-term trend is based on the Akaike information criterion (AIC). In this model, the value for the degrees of freedom corresponding to the minimum value of the AIC is the best *df* value. We choose 7 per year as the value for the degrees of freedom of the smoothing function of data variables.

## 4 Result

During this 5-year study, there were 221 hospitalizations for RA-ILD in Jinan city. Table 1 presents the descriptive statistics data for monthly hospital admissions due to RA-ILD in different sexes, age ranges, and monthly environmental statistics. The monthly average concentrations for PM_2.5_, PM_10_, SO_2_, NO_2_, and O_3_ were 66.9 μg/m^3^, 127.8 μg/m^3^, 28.2 μg/m^3^, 46.9 μg/m^3^, and 107.2 μg/m^3^, respectively.

**Table 1:** Descriptive statistics of monthly RA-ILD admissions, air pollutants and weather in Jinan, China (January 1^st^, 2015 to December 31^st^, 2019).

	Percentile					
Variable^∗^	Mean ± SD	Min	Max	P25	P50	P75
Total (*N* = 221)	3.68 ± 1.75	1.00	8.00	3.00	3.00	5.00
Sex						
Male (n = 92)	1.53 ± 1.21	0	5.00	1.00	1.00	2.00
Female (n = 129)	2.13 ± 9.17	1.00	5.00	1.25	2.00	3.00
Age						
<65 (n = 140)	2.33 ± 0.98	1.00	5.00	2.00	2.00	3.00
>65 (n = 81)	1.35 ± 0.97	0	4.00	1.00	1.00	2.00
Air pollutant concentrations (monthly average)						
PM_2.5_ (μg/m^3^)	66.90 ± 28.10	26.00	158.00	42.25	61.00	81.75
PM_10_ (μg/m^3^)	127.87 ± 40.71	53.00	232.00	98.00	124.00	152.50
SO_2_ (μg/m^3^)	28.15 ± 19.08	8.00	92.00	13.25	22.50	34.75
NO_2_ (μg/m^3^)	46.85 ± 11.85	26.00	73.00	35.50	46.50	55.75
O_3_ (μg/m^3^)	107.23 ± 47.84	27.00	206.00	64.00	108.00	144.75
Weather conditions (monthly average)						
Temperature(°C)	15.43 ± 9.84	−1.50	29.10	5.05	16.15	25.27
Humidity (%)	55.12 ± 11.52	35.70	82.00	46.30	54.70	62.78

Max = maximum value, Min = minimum value, P25 = 25th percentile, P50 = 50th percentile, P75 = 75th percentile, SD = standard deviation. n = number of observations.

∗ monthly average.

Spearman's correlation coefficients related to the air pollutants are shown in Table 2. The correlation between individual pollutants is reflected in Table 2. The results showed a positive correlation among PM_2.5_, PM_10_, SO_2_, and NO_2_. In contrast, there was an apparent negative correlation between the other pollutants and O_3_, and the results were statistically significant. (p < 0.01). Especially high correlations were found between PM_2.5_ and PM_10_ (R = 0.884), PM_2.5_ and SO_2_ (R = 0.881), PM_10_ and SO_2_ (r = 0.824), and O_3_ and NO_2_ (R = -0.843).

**Table 2:** Correlation coefficients between individual air pollutants.

Variable	PM_2.5_	PM_10_	SO_2_	NO_2_	O_3_
PM_2.5_	1.000	0.884^∗^	0.881^∗^	0.748^∗^	−0.731^∗^
PM_10_	–	1.000	0.824^∗^	0.733^∗^	−0.689^∗^
SO_2_	–	–	1.000	0.581^∗^	−0.556^∗^
NO_2_	–	–	–	1.000	−0.843^∗^
O_3_	–	–	–	–	1.000

∗ Statistically significant.

Table 3 reveals the estimated effects of air pollutants on hospitalizations for RA-ILD in single-pollutant models. The results showed that the levels of PM_2.5_, PM_10_, SO_2_ and NO_2_ were significantly associated with the number of admissions for RA-ILD. A tendency for higher O_3_ levels to be inversely associated with hospitalizations for RA-ILD was observed. PM_2.5_, PM_10_, and SO_2_ showed the strongest effect on the month (lag 0), and NO_2_ was most related to RA-ILD at a lag of two months (lag 2). The monthly admissions of RA-ILD increased by 0.875% (95% CI: 0.375–1.377%), 0.548% (95% CI: 0.148–0.949%), 1.968% (95% CI: 0.869–3.080%), and 1.534% (95% CI: 0.305–2.778%) for each 10 μg/m^3^ increase in PM_2.5_, PM_10_, SO_2_ and NO_2_, respectively.

**Table 3:** The effect of excess risk (ER) (95% CI) for hospitalization for RA-ILD per 10 μg/m^3^ increase in pollutant concentrations on the month of lag 0–2.

Variable	Lag0	Lag1	Lag2
PM_2.5_	0.875 (0.375,1.377)	0.355 (−0.120,0.832)	0.705 (0.188,1.224)
PM_10_	0.548 (0.148,0.949)	0.132 (−0.217,0.482)	0.401 (0.044,0.759)
SO_2_	1.968 (0.869,3.080)	0.478 (−0.212,1.174)	0.885 (0.148,1.634)
NO_2_	1.461 (0.262,2.675)	1.478 (0.272,2.699)	1.534 (0.305,2.778)
O_3_	−0.304 (−0.019, 0.589)	−0.379 (−0.085, 0.671)	−0.393 (−0.093, 0.694)

Table 4 shows that when exposed to high concentrations of PM_2.5_, PM_10_, NO_2_ and SO_2,_ the cumulative 0- to 2-month admissions for RA-ILD had the strongest effect.

**Table 4:** The effect of excess risk (ER) (95% CI) for hospitalization for RA-ILD per 10 μg/m^3^ increase in pollutant concentrations on the month of cumulative lag 0–2.

Variable	Lag01	Lag02
PM_2.5_	0.779 (0.222,1.338)	0.893 (0.304,1.485)
PM_10_	0.402 (−0.02,0.830)	0.541 (0.078,1.006)
SO_2_	1.347 (0.354,2.350)	1.568 (0.543,2.603)
NO_2_	1.806 (0.495,3.133)	1.917 (0.556,3.296)
O_3_	−0.383 (−0.088, 0.677)	−0.406 (−0.101, 0.710)

## 5 Discussion

This forward-looking epidemiological time-series study in Jinan, China, with documented exposure to air pollution, showed the correlation between air pollutants and hospitalization related to RA-ILD. We found that exposure to PM_2.5_, PM_10_, SO_2_, and NO_2_ aggravated the excess risk of hospitalization for RA-ILD. However, higher O_3_ tended to be inversely associated with hospitalization for RA-ILD. There is good reason to believe that lowering ambient air pollution concentration would lead to improvements in the health of people with RA-ILD.

No previous studies investigating the correlation between air pollutants and hospitalization for RA-ILD exist. Thus, we have performed an original study in this field, particularly for prolonged lag. Previous studies have mainly analyzed the correlation between air pollutants and RA or ILD of known or unknown etiology, although such studies are very limited, and the outcomes have not always been consistent.

Hart JE et al. found a 30% elevated risk of RA in participants whose homes were located near the road, who are more likely to be living within an environment with pollutants associated with traffic, revealing a possible etiological role of air pollution. ^[20]^ Similar studies have been conducted in Canada. Studies have shown that exposure to traffic-related pollutants such as PM_2.5_, NO, and NO_2_ increases the risk of rheumatoid arthritis. ^[21]^ The study also showed that O_3_ was associated with an increased risk of rheumatoid joints. Another case-control study showed a correlation between NO_2_ and SO_2_ exposure and the growing risk of RA, but there was no evidence of a growing risk of RA because of PM_10_. ^[22]^ In a recent 12-year case-control study in Korea involving 2220 subjects, O_3_ and CO exposure was positively correlated with the risk of RA in adults over 20 years of age.^[23]^ A retrospective cohort study from Taiwan Province showed that participants exposed to higher annual average pollutant concentrations of NO_2_ and PM_2.5_ had a growing risk of RA, and environmental factors may be a risk factor for rheumatoid arthritis. ^[24]^ However, the other 2 studies had contrary observations.^[25,26]^

The influence of air pollution is certain under numerous respiratory conditions, including poorly controlled asthma, ^[27]^ increased incidence of chronic obstructive pulmonary disease (COPD), ^[28,29]^ and respiratory-related mortality. ^[30]^ At present, there are also many studies showing the correlation between air pollutants and interstitial lung disease. Lucile Sesé et al. found that cumulative exposure to PM_10_ and PM_2.5_ increased the mortality of patients with IPF.^[31]^ Coralynn Sack et al. found that for every 40 ppb increase in NO_2_, the probability of developing ILD increased 1.77 times (95% CI from 1.06 to 2.95). Higher concentrations of pollutant exposure were associated with faster disease progression. ^[11]^ Christopher J et al. found that there was a close relationship between the level of PM_10_ and the decline in FVC in patients with ILD; for every 1 μg increase in PM_10_ in exposed patients, FVC decreased an additional 46 ml/year. ^[32]^

All the research above indicates a correlation between air pollutants and RA or ILD, and although it cannot prove an association between air pollutants and RA-ILD, we can hypothesize that air pollutants may also result in an increase in the incidence of RA-ILD through unknown mechanisms.

However, in our research, it was found that there was an adverse association between O_3_ exposure and the incident risks of RA-ILD. The correlation analysis between ambient air pollution showed a positive correlation between pm_2.5_, pm_10_, SO_2_, and NO_2_ and an apparent negative correlation with O_3_, and the results were statistically significant. (p < 0.01). The chemical coupling bond between O_3_ and NO_2_ in the atmosphere may explain the situation mentioned above. The degrees of exposure to O_3_ and NO_2_ are inextricably linked. O_3_ can be scavenged by traffic-produced NO_2_, and O_3_ is a harmful secondary pollutant produced by the reaction of volatile compounds in the troposphere with sunlight, which will lead to the consumption of NO_2_ and the accumulation of O_3_. ^[33]^ Higher exposure to NO_2_ always occurred with lower O_3_ exposure. That is, individuals who were often exposed to high levels of NO_2_ were also exposed to low levels of O_3_.^[34]^ This negative association could be the reason for the trend towards an adverse correlation between higher levels of O_3_ and lower incident risks of RA-ILD. In other words, O_3_ is not a protective factor for RA-ILD.

The risk factors for RA-ILD reported in previous studies include older age, male sex, smoking,^[35]^ exposure to occupational dust and the existence of multiple RA-related autoantibodies, including RF, ACPA, anti-heat-shock protein-90 (anti-HSP90) antibodies, ^[36]^ anti-PAD antibodies^[37]^ and anti-malondialdehyde-acetaldehyde adduct (anti-MAA) antibodies,^[38]^ Existing studies have found that smoking can promote the citrullination of proteins in the lungs, which can cause damage to the lungs and drive the development of RA.^[39]^ However, studies have also shown an increase in the citrullination of lung proteins in people who are not smokers. ^[40]^ This finding indicates that other substances can enter the lung from the outside through breathing, such as silicon dioxide, air pollutants and various microorganisms. They may have a mutual impact on genes and the immune mechanism to stimulate the production of ACPA or to promote the relevant immune response in patients with RA.^[14,41]^ This evidence is enough to show that environmental factors are crucial in the occurrence of RA-ILD.

The pathophysiology of RA-related lung diseases is complex and rare. According to the existing research, the concept of “gene-environment interactions with immune system interactions” is followed.^[42]^ Notably, some studies have shown that the lungs play a role in the mechanism of air pollutants affecting the pathogenesis of RA. Airway and alveolar epithelial cells are the first line of defense against inhaled substances, such as smoking-associated toxicants, air pollutants and pathogens, which are potential primary triggers of mucosal injury through the local lung and systemic oxidative stress and inflammation.^[43,44]^ Air pollutants inhaled in the respiratory tract, such as PM or gaseous pollutants, can stimulate neutrophils and mononuclear macrophages to release free reactive oxygen species, thereby activating nuclear factor kappa-B (NF-kB).^[45,46]^ NF-kB is a key regulator of pro-inflammatory cytokines in patients with rheumatoid arthritis. NF-kB can stimulate multiple cytokines, including tumor necrosis factor alpha (TNF-α), interleukin-1 (IL-1) and interleukin-6 (IL-6), to produce excess T helper lymphocyte type 1 (Th1) cells and induce a pulmonary inflammatory response. ^[46]^ There is persistent, amplified, chronic inflammation, which, in susceptible individuals with positive HLA-DRB1 shared epitopes, interacts with environmental factors. Protein citrullination occurs in the lungs, which further produces ACPA and then causes lung injury and clinical signs of RA. ^[47]^ Specifically, environmental exposure factors may provoke inherent immune cells, change the lung microenvironment, ^[14]^ and activate a series of proinflammatory cascade reactions and immune responses, resulting in interstitial lung disease or other lung injuries in patients with RA. This suggests that the lung is not only the target organ injured in RA but also may be an initiating site of the generation of antibodies in RA patients. Several studies have also confirmed that there are abnormal rheumatoid arthritis-related autoantibodies in the lung and serum before the onset of rheumatoid arthritis, which provides further evidence that the lung is a probable organ that generates autoimmunity related to RA before the onset of arthropathy. Demoruelle and his colleagues found that 65% of high-risk subjects with seropositivity and 86% of early rheumatoid arthritis subjects’ sputum were positive for no fewer than one antibody related to RA. Importantly, at least one rheumatoid antibody was also identified in 39% of high-risk patients whose serum was negative, supporting the idea that rheumatoid-associated autoantibodies can be produced locally in the lung. ^[48]^

There are several limitations to our study. First, hospital outpatients were excluded, and hospitalization was the only outcome measure in our study, which may underestimate the impact of exposure, as the actual population affected by exposure to air pollution would tend to be larger than that concluded by us. Second, data on ambient pollutant concentrations in our time-series analysis were gathered from the average daily data of fourteen fixed-site monitoring stations, which may not accurately reflect the actual pollutant exposure in the participants’ environment. Individual exposure levels may be far-ranging on the basis of many elements, such as living and job locations, physical activity, time spent outside, and vacations; our study cannot estimate the duration and magnitude of air pollution exposure on an individual patient level. Third, multiple air pollutants such as PM, SO_2_, NO_2_, NO, CO, O_3_ and some organic compounds have previously been associated with hospitalization for various diseases. However, only five of common pollutants were studied in my research due to limitations in the data collection phase. More comprehensive studies are needed to explore the relationship between them. Fourthly, the results may be influenced by the low statistical power attributable to the few incidences of RA-ILD in our research. Last, our study was conducted in only one district (Jinan, China), which ignores the variability of exposure to various air pollutants. Therefore, our results apply only to areas with similar pollution levels and environmental statuses.

Despite these limitations, we used time-series analysis to achieve a personal evaluation of exposure to air pollution and surveyed the effects of various lag times of exposure. We were the first to research the correlation between air pollutants and hospitalization due to RA-ILD.

In summary, we found that an increased number of hospitalizations for RA-ILD was associated with long-term exposure to air pollutants, including PM_2.5_, PM_10_, SO_2_, and NO_2_. These findings provide supporting evidence that ambient air pollutants might be a potential risk factor for RA-ILD. Despite the positive results presented here, we could not entirely give evidence of a clear mechanism to explain pollutant effects on RA-ILD. Further studies are needed to investigate the potential biological pathways underlying these associations to help identify potential contributing factors to the morbidity or progression of RA-ILD.

## Acknowledgments

To the members of the participating hospitals and patients.

## Author contributions

YFH, BJL and LY contributed to the study conception, analysis plan, supervised data analysis and revised the drafted paper .BJL and SGZ cleaned and analysed the data, drafted and revised the paper; All authors approved the submitted version of this paper.
